# Constructive Approaches for Understanding the Origin of Self-Replication and Evolution

**DOI:** 10.3390/life6030026

**Published:** 2016-07-13

**Authors:** Norikazu Ichihashi, Tetsuya Yomo

**Affiliations:** 1 Department of Bioinformatics Engineering, Graduate School of Information Science and Technology, Osaka University, 1-5 Yamadaoka, Suita, Osaka 565-0871, Japan; ichihashi@ist.osaka-u.ac.jp; 2 Graduate School of Frontier Biosciences, Osaka University, 1-5 Yamadaoka, Suita, Osaka 565-0871, Japan

**Keywords:** constructive approach, self-replication system, evolution, in vitro system

## Abstract

The mystery of the origin of life can be divided into two parts. The first part is the origin of biomolecules: under what physicochemical conditions did biomolecules such as amino acids, nucleotides, and their polymers arise? The second part of the mystery is the origin of life-specific functions such as the replication of genetic information, the reproduction of cellular structures, metabolism, and evolution. These functions require the coordination of many different kinds of biological molecules. A direct strategy to approach the second part of the mystery is the constructive approach, in which life-specific functions are recreated in a test tube from specific biological molecules. Using this approach, we are able to employ design principles to reproduce life-specific functions, and the knowledge gained through the reproduction process provides clues as to their origins. In this mini-review, we introduce recent insights gained using this approach, and propose important future directions for advancing our understanding of the origins of life.

## 1. Introduction

There must have been many hurdles to overcome before complex living things arose on the early earth. The first hurdle was the birth of organic compounds. If the first life appeared in the hypothetical RNA world [1], the components of RNA, i.e., ribonucleotides, must have been created abiologically beforehand. Similarly, if the first life depended on proteins, amino acids must have existed in advance. Many studies have been published focusing on the abiological generation of these organic compounds (recently reviewed in [2]). The second hurdle was the assembly of organic compounds to form polymers that can work as information storage molecules, perform enzymatic activities, or carry out other biological functions. The appearance of enzymatic activity from a random pool of polymers is plausible, considering that ligand-binding activities often appear from random sequences of nucleotides or amino acids in a small, lab-scale population [3,4,5,6,7]. The third hurdle was the appearance of a molecule, or a set of molecules, with self-replicating activity, such as a hypothetical self-replicating ribozyme. However, the appearance of a single kind of self-replicating molecule still does not guarantee the evolution of a complex life form. Complex living organisms rely on the cooperation of many different molecules, such as polynucleotides, proteins, carbohydrates, and lipids. Before the appearance of a primordial precursor to complex cellular life, the fourth hurdle would have had to be overcome—many kinds of molecules needed to work in a concerted manner for the growth and maintenance of the whole. The definition of life is ambiguous [8,9]; nevertheless, many people would agree that one of the requirements for life is the ability to evolve and thus to produce the diversity of the modern living world. The acquisition of the ability to evolve was the fifth, and perhaps the most difficult, hurdle that had to be overcome during the origins of life.

How can a set of molecules achieve self-replication, and how can such molecules then acquire the ability to evolve? A direct strategy for finding the answers to these questions is to construct an evolvable, self-replicating system in a test tube. This type of strategy is called the “constructive approach” [10,11], in which a target biological function is constructed using known molecules. This strategy is also sometimes called the “semi-synthetic approach” [12,13,14], because the components are not restricted to abiologically synthesized molecules, and can include biomolecules purified from living things. Through this approach, we can define conditions that are sufficient for a set of molecules to acquire a given target biological function. This knowledge will contribute to the understanding of possible scenarios for the emergence of life on earth. In this mini-review, we introduce several achievements made in the field using the constructive approach, focusing on the ability of experimental systems to self-replicate and evolve. Finally, we present possible future experimental directions.

## 2. What Is the Constructive Approach?

The constructive approach can be defined as an approach that attempts the construction of a target biological function, using defined molecules, in order to understand the conditions required to achieve that function. The key point in this approach is that it uses a defined set of molecules, rather than a complex mixture such as a crude cell extract. This is because complex mixtures make it impossible to identify important components and interactions required for the target function. The molecules used are not limited to those that could have existed on the ancient earth, and any nucleotide (DNA and RNA) or amino acid polymers can be used. However, the fact that a set of molecules is experimentally sufficient to achieve a target biological function does not simply imply that the same set of molecules was actually involved in achieving that biological function during the origin of life on earth. Therefore, this approach only proposes plausible physicochemical routes toward the origin of life.

## 3. Construction of Self-Replicating Systems

**Figure 1:**
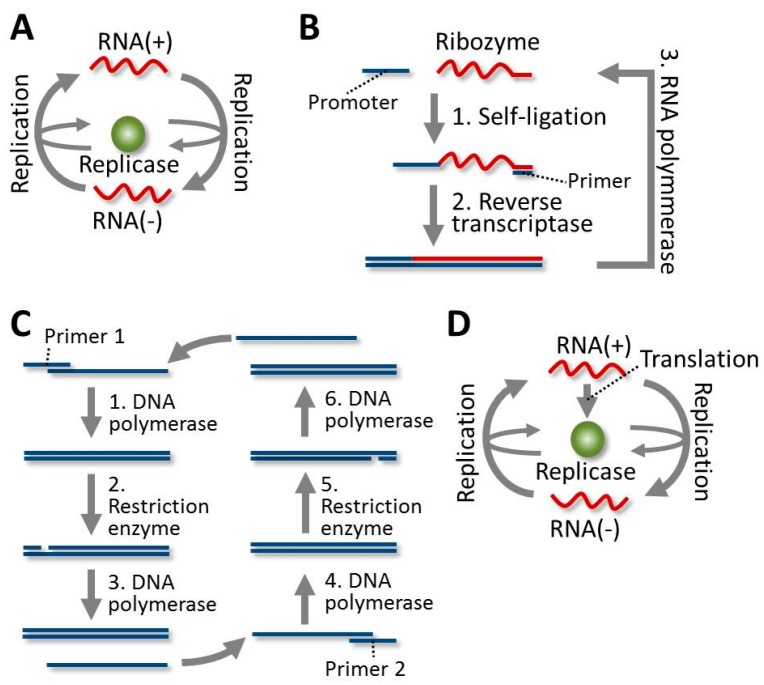
Self-replication schemes. RNA and DNA are colored red and blue, respectively. A schematic of Spiegelman’s RNA system is shown in (**A**). The complimentary strand RNA (RNA (−)) is synthesized from the template RNA (RNA (+)) by an RNA replicase. The template RNA is then resynthesized from the complimentary strand by the replicase. (**B**) The ribozyme-based self-sustained sequence replication (3SR) system is shown. First, the template RNA (a ribozyme) catalyzes its own ligation to a DNA containing promoter sequence (1). Then, a DNA primer hybridizes to the 3′ terminus, and the complementary RNA is synthesized by a reverse transcriptase (2). Finally, the original template RNA is transcribed from the RNA/DNA hybrid by a DNA-dependent RNA polymerase (3). (**C**) A schematic of the strand displacement amplification (SDA) system is shown. Primer 1 first binds to the single-stranded template DNA. DNA polymerase then elongates both strands (1). Next, an endonuclease introduces a nick in one of the strands (2). DNA polymerase synthesizes a DNA strand from the nick, and displaces one of the strands, which then binds to Primer 2 (3). DNA polymerase then generates double-stranded DNA using Primer 2 and this DNA strand (4). Next, another endonuclease introduces a nick in the resulting double-stranded DNA (5). Finally, DNA polymerase reproduces the original single-stranded template DNA by synthesizing a strand starting from the nick (6). (**D**) The translation-coupled RNA replication (TcRR) system is shown. This system is composed of a template RNA that encodes an RNA replicase, along with all translation factors, tRNAs, ribosomes, NTPs, amino acids, and so on. The RNA replicase is translated internally, and it then replicates the original template RNA as in Spiegelman’s system (**A**).

Various types of self-replicating systems have been constructed from defined molecules. The oldest example is a self-replicating RNA system developed by Spiegelman’s group in 1967 [15]. They constructed a self-replicating system from the RNA genome of the bacteriophage Qβ that used its RNA-dependent RNA-polymerase (RNA replicase) (Figure 1A). In this system, the RNA polymerase synthesized the complementary strand using the template, and RNA polymerase then reproduced the original template from the complementary strand. Another example was self-sustained sequence replication (3SR), a system in which the template RNA polymers replicated themselves through a DNA stage by using a set of proteins: a reverse transcriptase, a DNA polymerase, an RNA polymerase, and an RNA degradation enzyme [16]. The 3SR system has also been modified to depend on ribozyme activity from the template RNA [17] (Figure 1B). A simpler DNA self-replication system has been constructed called the double-strand displacement amplification (SDA) system. In the SDA system, the template DNA is replicated through successive processes of strand-displacement polymerization from a nick in the template DNA [18] (Figure 1C). In all of the above-mentioned self-replicating systems, the polymerases were externally supplied by researchers. In contrast, in the translation-coupled RNA replication (TcRR) system, the RNA-dependent RNA polymerase was translated from the template RNA [19] (Figure 1D). In the TcRR system, the template RNA had competing dual roles as both the replication template and the translation template. Since a single molecule of template RNA could not simultaneously work with the RNA replicase for replication and the ribosome for translation, the coordination of these dual roles was important for the TcRR system [20].

Several types of protein-free self-assembling systems have been constructed. Joyce’s group has constructed a self-replicating RNA system by using a self-recombinase ribozyme and its fragments [21]. Lehman’s group developed a cooperative self-replicating network using the fragments of other types of self-ligating ribozymes [22]. Furthermore, a ribozyme with RNA polymerization activity has been developed, although complete self-replication has not been achieved [23,24]. These RNA-based, protein-free replication systems provide strong evidence supporting the RNA world hypothesis [25]. Conversely, Ghadiri’s group has constructed a peptide-based self-replicating system, in which a peptide containing a leucine-zipper domain catalyzes amide-bond formation between smaller peptides to synthesize a copy of itself [26,27]. Luisi’s and Sugawara’s groups have reported vesicle-based self-reproducing systems that used artificial lipids [28,29,30]. These vesicles catalyze the formation of their own components, thus growing and eventually splitting to produce daughter vesicles. Sugawara’s group has further reported the coupling of the growth of a vesicle with an internal DNA replication system [31]. Much simpler autocatalytic chemical reactions have also been reported [32,33,34,35]. These examples of self-replicating systems imply that self-replication-type reactions could have occurred relatively easily if the appropriate sets of molecules existed.

## 4. Construction of the Ability to Evolve

Do the self-replicating systems mentioned above have the ability to evolve? For a molecular system to evolve, it must satisfy three requirements [36]:

1. Replication
2. Inheritable variation
3. Selection

Among the self-replicating systems mentioned, only Spiegelman’s RNA system, the SDA system, the 3SR system, and the TcRR system satisfied all three requirements. In these systems, inheritable variation was produced through the misincorporation of nucleotides into the RNA or DNA during polymerization. When replication in these systems was continued for many generations, mutant RNAs or DNAs that replicated faster came to dominate the population. This was the result of selection within these systems favoring faster replicating molecules. In contrast, there was no inheritable variation within the other self-replicating systems. This was because in protein-free self-ligating ribozyme systems, peptide systems, and vesicle systems, the molecules were not synthesized through polymerization. In this section, we describe in detail the evolution of the DNA and RNA within the four nucleotide-based systems. It is notable that in the field of directed evolution, there have been many studies using artificial selection methods, such as the systematic evolution of ligands by exponential enrichment (SELEX), to drive evolution in vitro [37,38,39,40]. However, in this mini-review we focus only on studies that used selection based on the number of directly produced offspring, because only this type of selection could have been relevant to early life on earth.

### 4.1. Evolution of the RNA in Spiegelman’s RNA System

Spiegelman’s system contained the template RNA and the purified RNA replicase from bacteriophage Qβ. During this replication process, mutations and deletions were introduced to the RNA through replication errors, and non-homologous recombination, respectively [41]. Spiegelman’s group continued this replication reaction for many generations by successively transferring aliquots from each reaction to a new mixture that contained the same ingredients. They continued the transfer experiment for 74 rounds, and found that the replication amount per round of reaction gradually increased, whereas the size of the template RNA gradually decreased [15]. The final RNA had lost all three genes that were originally encoded, but kept the recognition sequence for the RNA replicase. Due to its smaller size, this final RNA replicated much faster than the original RNA, and thus became the dominant molecule within the population.

Ethidium bromide resistance was another example of evolution demonstrated using Spiegelman’s RNA system. The transfer experiment was repeated in the presence of a small amount of ethidium bromide, which partly inhibits RNA replication. They found that after 25 rounds of transfer, the template RNA had acquired three-point mutations, and was more resistant to ethidium bromide than the original RNA [42]. These two experiments clearly demonstrated that Darwinian evolution could occur in vitro, as long as the requirements mentioned earlier were satisfied.

### 4.2. Evolution of the RNA in the 3SR System

In the 3SR system, the template RNA replicated via a DNA stage by using a reverse transcriptase and an RNA polymerase. During this process, mutations were incorporated through polymerization errors, and they were then inherited by all subsequent templates. Therefore, if a mutant RNA with higher replication ability appeared, it should have dominated the population after many rounds of replication. Wright and Joyce performed 300 rounds of serial transfer experiments using the ribozyme-based 3SR system (Figure 1B). The resulting dominant RNA sequence had acquired 15 mutations and 10^4^-fold higher self-ligation activity [43]. In another experiment using the ribozyme-based 3SR system, a small RNA named RNA-Z dominated the population after only few hours of reaction [17]. RNA-Z did not have self-ligation activity, but replicated through a quicker, alternative process. This interesting example showed that a replication system with the ability to evolve could spontaneously produce novel and unexpected types of self-replication.

### 4.3. Evolution of the DNA in the SDA System

The template DNA in the SDA system (Figure 1C) can also evolve, because inheritable mutations are introduced during polymerization. Walter and Strunk performed 100 rounds of the SDA reaction, and observed that the size of the template DNA gradually decreased, because a smaller DNA replicated faster and needed fewer nucleotide monomers [44]. This evolution of a smaller template was the same phenomenon observed in Spiegelman’s RNA system. Analysis of the sequences throughout the course of the experiment revealed that several deletions in different regions successively appeared, and their frequency then increased within the population.

### 4.4. Evolution of the RNA in the TcRR System and the Requirement of a Compartment

**Figure 2:**
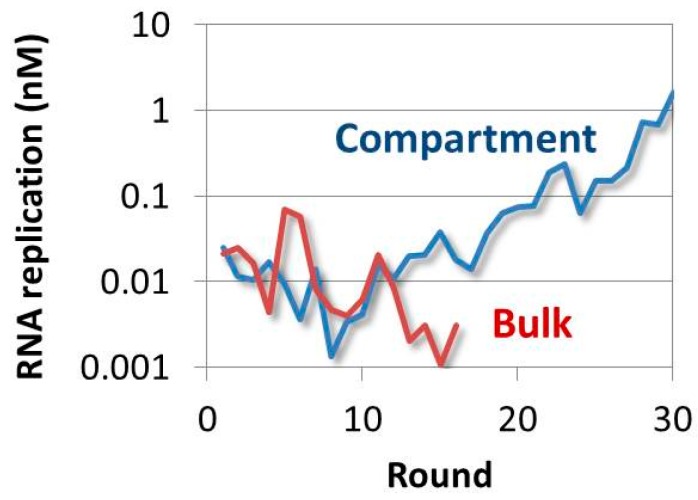
The effect of compartmentalization on replication in the TcRR System. We measured the template RNA concentration for each round of the transfer experiments. Under bulk conditions without compartmentalization, the template RNA was not detected after 17 rounds of a transfer experiment (red line). Conversely, under compartmentalized conditions, RNA replication continued and then increased due to evolution (blue line). Only part of the result is shown here. Reproduced with permission from Nature Publishing Group [45].

**Figure 3:**
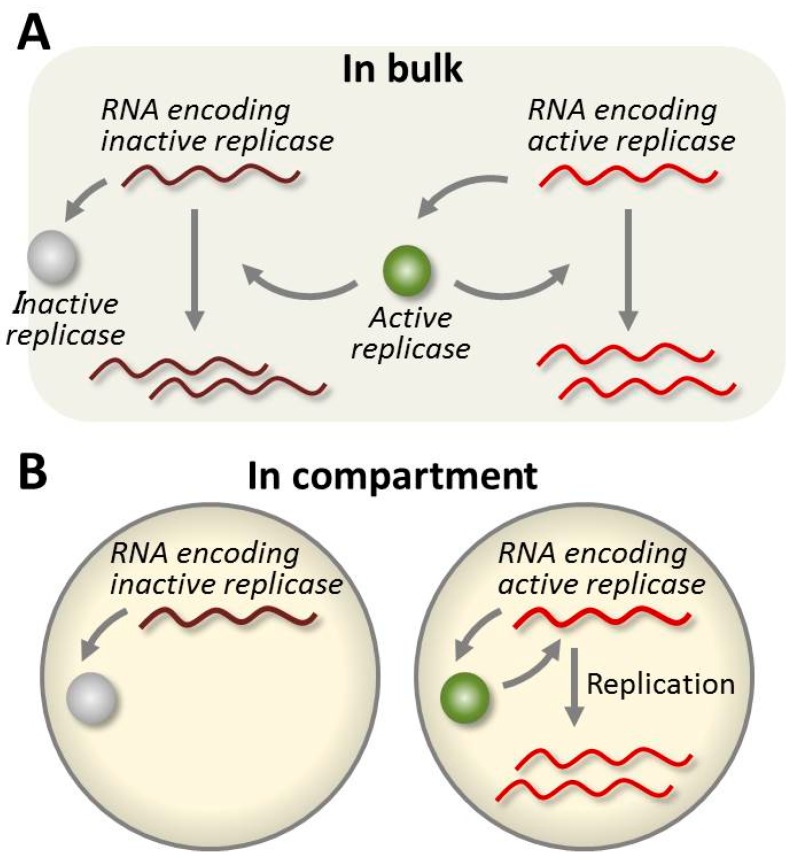
Schematic drawing of the TcRR system under bulk or compartmentalized conditions. (**A**) Under bulk conditions, the replicases are shared among all the template RNA, and thus even a mutant template RNA that encodes inactive replicase can replicate using an active replicase translated from another template RNA. (**B**) Under compartmentalized conditions, each template RNA can use only the replicase translated from itself, and thus a mutant template RNA that encodes an inactive RNA replicase cannot replicate.

In the evolution of Spiegelman’s self-replicating RNA, the template RNA became smaller and lost all its originally encoded genes (see Section 4.1). This was because Spiegelman’s system did not contain translation machinery, and thus the encoded genes were just a burden for replication. To incorporate translation machinery, our group combined Spiegelman’s system with a cell-free translation system to construct the TcRR system, wherein the RNA replicase was translated from the template RNA (Figure 1D). We first performed a transfer experiment for 17 rounds under commonly used, non-compartmentalized conditions [45]. In the TcRR system, mutations were introduced and inherited as in Spiegelman’s system, and therefore, the RNA was expected to evolve. However, we did not observe evolution of the RNA; instead, the replication per round decreased and became undetectable after 17 rounds (Figure 2). This reduction of replication was caused by a lack of negative selection against the mutated RNAs that encoded inactive replicases. Most random mutations inactivated the replicase gene, but the RNAs encoding inactive replicases replicated as well as the original RNAs encoding active replicases by using active replicases translated from the original RNAs (Figure 3A). Therefore, as the frequency of the mutant RNAs harboring inactive replicases increased with the accumulation of random mutation on the replicase genes, the replication rates of all of template RNAs in the experiment decreased.

Compartmentalization of the reaction solution solved this problem. If the reaction was compartmentalized so as to have one RNA molecule per compartment, each template RNA could only use the replicase translated from itself (Figure 3B). Under such conditions, a mutant RNA that encoded an inactive RNA polymerase would not replicate, and would be competed out of the population (i.e., negatively selected). Therefore, compartmentalization was required for the RNA to continuously self-replicate and evolve in the TcRR system. This additional requirement appeared because the phenotype (replicase activity) should exclusively contribute to the amplification of the corresponding genotype (RNA sequence). The phenotype and genotype were connected by the addition of compartmentalization.

We encapsulated the TcRR system into a water-in-oil emulsion with droplet sizes of approximately two micrometers in diameter, and performed the transfer experiment for 128 rounds [45]. In contrast to the results under the conventional in vitro condition without compartmentalization, the replication amount per round increased after eight rounds, and after 30 rounds it was more than 100-fold higher than the initial amount per round (Figure 2). The final RNA after 128 rounds had accumulated 38 mutations, but remained almost the same size, and retained an active replicase gene. This result demonstrated that compartmentalization was another requirement for the evolution of the RNA in the TcRR system. We also performed the same transfer experiment under another condition where the translation rate of the replicase was decreased by reducing the concentration of ribosomes [46]. In this case, the resulting template RNA had an altered sequence around the ribosome-binding site that increased translation. This experiment demonstrated that the template RNA in the TcRR system had the ability to adapt to different environmental pressures.

Other than providing a phenotype to genotype linkage, compartmentalization has another role in the TcRR system; it provides protection against selfish or parasitic replicators. We found that a small RNA often appeared spontaneously in the TcRR system during the uncompartmentalized reaction. The small RNA was generated from the template RNA, probably through recombination [41,47]. The small RNA had lost the replicase gene, but retained the recognition sequence for the replicase. Therefore, the small RNA rapidly replicated by exploiting the RNA polymerase translated from the template RNA. In this sense, the small RNA was parasitic. The parasitic RNA replicated much faster than the template RNA due to its small size, and eventually competitively inhibited template RNA replication under these conditions. In experiments with compartmentalization, the spontaneously appearing parasitic RNA was confined to a minor fraction of the compartments, while the template RNA in other parasite-free compartments replicated normally [47]. Therefore, compartmentalization has another role as a shelter from parasitic replicators. Furthermore, compartmentalization is reported to have another positive effect on internal reactions through concentrating the molecules involved (recently reviewed in [48]). The experimental evidence suggests that compartmentalization in a cellular structure might have played several essential roles in the emergence of life.

## 5. Analysis of the Evolutionary Process Using an in Vitro Self-Replicating System

One advantage of in vitro self-replicating systems is their simplicity (the small size of the genetic material). This enables detailed analysis of the evolutionary process at a level impossible when using complex organisms. One example is the drawing of a fitness landscape; the simplicity of the in vitro self-replication systems enables the depiction of their fitness landscapes at higher resolution. A fitness landscape is a visual representation of the relationship between genotype and fitness, and it is an important tool for understanding evolution in a system [49]. In these landscapes, fitness is represented as the height in sequence space, and evolution is represented as a climbing process along the landscape. Although the fitness landscape has been an abstract concept for a long time [49], recent advances in sequencing technology allow partial drawing of a landscape based on experimental data (reviewed in [50,51]). As compared to the genomes of natural cells, the much smaller, more easily sequenced RNAs and DNAs of in vitro self-replicating systems provide a significant advantage for the analysis of their fitness landscapes. In the following parts of this section, we describe our study of the fitness landscape of the RNA in the TcRR system. This serves as an example of how such analyses can allow for better understanding of the evolutionary process.

**Figure 4:**
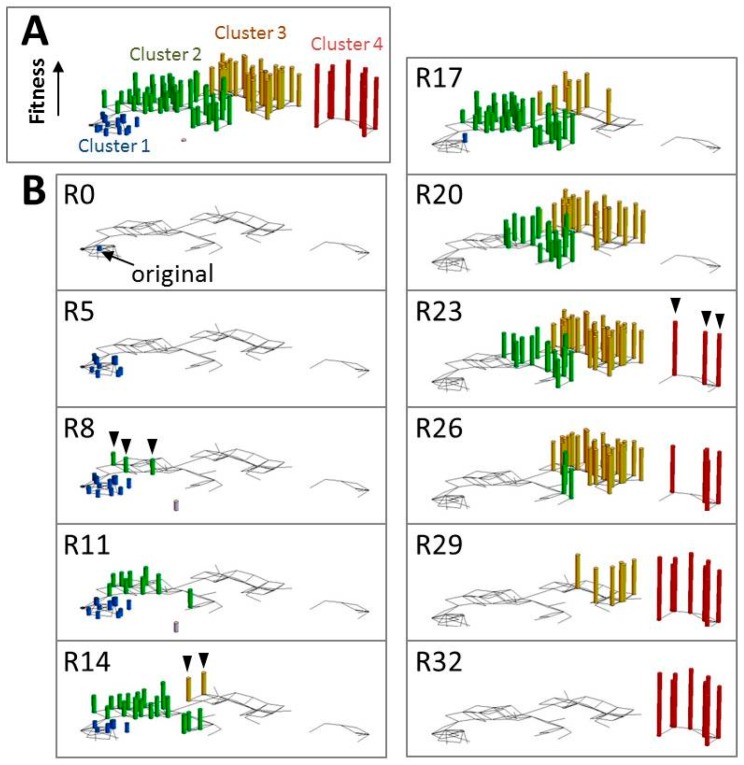
The evolutionary process of the template RNA in the TcRR system. (**A**) The template RNA sequences were analyzed over 32 rounds (R1 to R32) of a transfer experiment using the TcRR system. The Hamming distances of 91 major genotypes were calculated and projected on a two-dimensional landscape. The genotypes were classified into four major clusters (Clusters 1 to 4) and colored accordingly. The fitness of each genotype was estimated from the rate of change in its frequency and plotted on the *z*-axis. (**B**) Only the genotypes that constituted more than 1% of the population at each round were shown. The RNA population first diverged within Cluster 1 (R0 to R5). Three RNA genotypes that had higher fitness then appeared within Cluster 2 (arrowheads in R8). The RNA population then continued to diverge within Cluster 2 (R8 to R11). Subsequently, two genotypes that had much higher fitness appeared in Cluster 3 (R14, arrowheads). The RNA population again diverged within Cluster 3 (R14 to R20), and three new genotypes appeared in Cluster 4 (R23, arrowheads). Finally, the population diverged within Cluster 4 (R23 to R32). Reproduced with permission from Oxford Journals [52].

We sequenced the RNA of more than 200 clones at 11 points in the first 32 rounds of a TcRR transfer experiment using a next-generation sequencing technology [52]. We observed several trajectories for mutations in the RNA population; some mutations increased their frequency constantly and became fixed in the population, whereas others increased their frequency temporarily and then disappeared due to clonal interference [53]. During this evolutionary process, we found 91 different genotypes that consisted of 22 mutations, and these genotypes formed four clusters in sequence space (Figure 4A). Because the *z*-axis in Figure 4 shows the fitness (replication rate), this figure represents part of the fitness landscape. To visualize the trajectory of each genotype, we showed the major genotypes (more than 1% of the population) as bars at each round of the transfer experiment (Figure 4B). The evolutionary process showed a repeating pattern of diversification in the sequence space (e.g., rounds 0 to 5, 8 to 11, and 14 to 20) followed by the appearance of mutants in the next cluster (arrowheads in rounds 8, 14, and 23). The resulting mutants then dominated the population in the following rounds. This repeating process of diversification and domination by mutants with higher fitness is exactly the process of Darwinian evolution [54]. Presently, we do not know whether this landscape and evolutionary process is specific to the TcRR system, or occurs in other systems, because there have only been a limited number of analyses [50]. Further studies of in vitro self-replicating systems may reveal characteristics of the evolutionary process that are applicable to the natural world.

## 6. Lessons from the Constructive Approach

In the previous sections, we have introduced several types of in vitro self-replication systems and described evolution within them. What have we learned from these experiments? First, we have learned that the ability to evolve is not a characteristic unique to living things. Transfer experiments using several systems have demonstrated that a set of molecules will autonomously evolve as long as it satisfies three requirements (self-replication, inheritable variation, and selection based on the number of offspring). The relatively easy reproduction of evolution in vitro is consistent with the possibility of an evolvable system having spontaneously formed on the ancient earth.

The second lesson is that the evolutionary pattern differs among the self-replication systems. In the cases of Spiegelman’s RNA system and the SDA system, the sizes of the templates gradually decreased during evolution. In the cases of the ribozyme-based 3SR system and the TcRR system, the original template sizes were maintained, and the point mutations became fixed within the population. This difference is explained by the roles of the template RNA or DNA. The polynucleotides in Spiegelman’s system and the SDA system are only replication templates, and thus reducing their size is beneficial because smaller templates can be replicated faster. In contrast, to replicate themselves the templates of the ribozyme-based 3SR system and the TcRR system must encode an active self-ligating ribozyme or an active replicase, respectively. Therefore, large deletions that destroy the encoded ribozyme or gene do not occur, but point mutations are accumulated. 

The third lesson is the importance of compartments for the evolution of multi-component self-replicating systems such as the TcRR system. This requirement suggests that a primitive life form composed of multiple molecules must have had a cellular structure to acquire the ability to evolve, and it implies that cellular structures may date back to the early origins of life. The size of a cellular structure is also important. The sizes of the first cellular structures would have depended on the concentrations and affinities of the internal molecules. For example, in the case of an extant protein with a high affinity for polynucleotides (Kd ~1 nM), one molecule is enough for the concentration to become more than the Kd value in a 1-µm-diameter compartment, while 10^3^ or 10^6^ molecules of the protein are required to reach the same concentration in 10- or 100-µm-diameter compartments, respectively. If the affinity of an ancient protein for its ligand were lower, the required number of protein molecules would increase. Because ancient translation machinery was likely inefficient, a smaller size (ideally less than 1 µm diameter) would have been better for a primitive cell. Several cell-like compartments are reported to have possibly existed on the ancient earth, such as fatty acid vesicles [55] and ice crystals [56] (reviewed in [57]). Future studies concerning the possible sizes of compartments would be important.

## 7. Future Directions

In this mini-review, we introduced several examples of the constructive approach, focusing on self-replication and the ability to evolve. What is the next evolutionary hurdle for these self-replicating systems to overcome? One possible hurdle is the evolution of complexity. In the case of the TcRR system, only one replicase gene is encoded on the template RNA. Future template RNAs must encode more genes and acquire more complex replication systems to simulate the next stages of the early origin of life. In the case of the TcRR system, the largest problem is the severe structural restriction on the template RNA. Biochemical analysis reveals that a template RNA that is continuously replicable with the bacteriophage Qβ RNA replicase must have a strong secondary structure throughout [58]. Otherwise, it forms a double-stranded RNA after replication by hybridizing with the newly synthesized complementary strand, and the resulting double-stranded RNA cannot be a template for replication or translation [59]. This requirement of secondary structure places a severe restriction on the possible sequence of encoded genes, and makes the addition of another gene to the template RNA difficult. The problem of double-stranded RNA formation is not unique to the TcRR system, but applies to all bacteriophages and most fungal viruses that have single-stranded RNA genomes [58]. Interestingly, the genomes of single-stranded RNA viruses of other organisms, such as vertebrates, escape this problem by some unknown mechanism. By learning from these viruses, it might be possible to design a more complex version of the TcRR system.

Another direction is the introduction of the translation system into DNA-based self-replicating systems, such as SDA, in which the template double-stranded DNA is replicable without structural requirements. Some translation-coupled self-replicating DNA systems have been reported. These include a system based on genome replication in *Escherichia coli* [60] and a system based on rolling-circle type replication [61], although recursive replication has not been achieved in either case. The construction of a translation-coupled recursive self-replicating system is one of the next important challenges. 

The development of a protein-free self-replication ribozyme is another important challenge. Although complete self-replication has not been achieved, recent advances in the past few decades have allowed for superior RNA polymerization activity of ribozymes [23,24]. In the near future, it may be possible to develop a self-polymerizing ribozyme, which will be strong supporting evidence for the RNA world hypothesis [1].

Several other aspects of cellular function have been reproduced in vitro, although the efficiencies achieved have not yet been comparable to those in a living cell. These aspects include the growth and division of fatty acid vesicles or more complex liposomes [62,63,64,65], gene expression networks [66], and a mechanism to react to the extracellular environment [67]. The next important challenge is combining these functions with a self-replicating system of DNA or RNA to synthesize a more cell-like system. For that purpose, we must figure out how to coordinate the reproduction of cellular structures with DNA or RNA self-replication. Such artificial cell systems would provide experimental evidence of possible scenarios for the origin of complex life on earth.
